# Effects of Attractants on the Growth Performance, Antioxidant Capacity, Immunity, and Histology of Largemouth Bass Larvae (*Micropterus salmoides*)

**DOI:** 10.1155/anu/9641984

**Published:** 2025-12-14

**Authors:** Jianle Yang, Haoze Wang, Xiaorui Fan, Jiaqi Wang, Jianhua Zhao, Qiyou Xu

**Affiliations:** ^1^ College of Life Science, Huzhou University, Huzhou, 313000, China, zjhu.edu.cn; ^2^ National Local Joint Engineering Laboratory of Aquatic Animal Genetic Breeding and Nutrition, Huzhou, 313000, China; ^3^ Zhejiang Provincial Key Laboratory of Aquatic Bioresource Conservation and Development Technology, Huzhou, 313000, China

**Keywords:** attractants, growth performance, intestinal health, larvae, *Micropterus salmoides*

## Abstract

This study evaluated the effect of four attractants on the growth performance, antioxidant capacity, immunity, and histology of largemouth bass larvae (*Micropterus salmoides*). A total of 75,000 larvae (~1.25 mg) were randomly divided into 15 tanks (five groups with three replicates), with 5000 larvae per tank. The experimental diets were isonitrogenous (54.45% crude protein) and isolipidic (13.20% crude lipid), including a control group (CON, no attractants), taurine (TAU) group (8 g/kg, trimethylamine oxide (TMAO) group (0.25 g/kg), dimethyl‐β‐propiothetin (DMPT) group (5 g/kg), and nucleotides (NTs) group (0.8 g/kg). The feeding trial was conducted in tanks (40 cm × 60 cm × 80 cm) for 21 days. The larvae were fed with *Artemia* three times daily for 7 days. The 21‐day feeding trial started on the 8th day, alternated with feed six times daily from the 8th to 15th day, then fully transitioned to experimental diets with adjusted feed sizes. The results showed that TAU, DMPT, and NT groups significantly increased the final body weight (FBW), weight gain (WG), and specific growth rate (SGR) (*p* < 0.05), while TMAO, DMPT, and NT groups significantly increased the survival rate (SR) (*p* < 0.05), compared with the CON group. During the air stress challenge, compared with the CON group, all treatments significantly prolonged survival time (*p* < 0.05). Compared with the CON group, the TAU and NT groups significantly increased the activities of superoxide dismutase (SOD), catalase (CAT), and glutathione (GSH) content, and decreased the malondialdehyde (MDA) content (*p* < 0.05), and the total antioxidant capacity (T‐AOC) in the NT group was significantly increased (*p* < 0.05). All treatments significantly increased the activities of acid phosphatase (ACP) and alkaline phosphatase (AKP; *p* < 0.05). Compared with the CON group, the histological intestine indicated that all treatments significantly increased the intestinal fold height and decreased the muscular layer thickness, crypt depth, and fold width (*p* < 0.05); the histological liver indicated that the TMAO and NT groups could improve liver cells. In conclusion, all attractants exerted positive effects on largemouth bass larvae, with TAU, DMPT, and NT demonstrating significant growth enhancement associated with improved antioxidant capacity, immune response, and tissue histology. Further research is needed to clarify mechanisms and optimal doses.

## 1. Introduction

The feeding behavior of fish is an important foundation for their survival and reproduction, involving the processes of seeking, identifying, capturing, and digesting food, providing the material and energy basis for their survival, growth, and reproduction [1]. In recent years, in order to improve the palatability of fish feed and reduce the dependance on live baits, attractants are often added to the feed, thereby reducing feed waste and increasing aquaculture productivity [2]. The results demonstrated that many attractants can stimulate the appetite of fish and improve their feeding efficiency, such as taurine (TAU), trimethylamine oxide (TMAO), dimethyl‐β‐propiothetin (DMPT), and nucleotides (NTs) [3–6]. TAU, a sulfur‐containing amino acid [6], has been found to enhance the survival rate (SR), growth performance, antioxidant capacity, and stress resistance in fish, such as common carp (*Cyprinus carpio* L.) [7], gilthead seabream (*Sparus aurata*) [8], and Nile tilapia (*Oreochromis niloticus*) [9]. TMAO, a biogenic amine with a unique fresh and sweet taste, has been proven to be an important feeding attractant for fish, such as rainbow trout (*Oncorhynchus mykiss*) [5], and taimen (*Hucho taimen*) [10]. DMPT has been shown to promote appetite and enhance antioxidant capacity, such as in grass carp (*Ctenopharyngodon idella*) [11] and GIFT tilapia (*Oreochromis* sp.) [12]. NTs, low‐molecular‐weight compounds, play a crucial role in genetic information, energy metabolism, and cell signal transduction [13], and their addition to the diet can improve the growth performance of fish and enhance intestinal morphology and function, such as Chinese perch (*Siniperca chuatsi*) [4], meager (*Argyrosomus regius*) [14], and European seabass (*Dicentrarchus labrax*) [15]. Despite existing studies on feeding attractants in aquatic animals, further larva‐specific studies specifically on larvae are necessary.

The larval stage represents the most vulnerable phase in the fish life cycle [16]. In fact, existing studies have confirmed that the addition of suitable phagostimulants during the larval stage can activate early sensory receptors. This activation not only guides larval fish to actively locate and ingest feed but also promotes the secretion of digestive enzymes and improves intestinal development, and helps larval fish to pass through key developmental stages such as the “first‐feeding stage” and “metamorphosis stage.” For instance, in the rearing of larvae of marine fish species including *Ompok bimaculatus* and large yellow croaker (*Larimichthys crocea*), supplementation with amino acid‐based phagostimulants or NT has increased SRs by 15%–20% [17, 18]. Overall, there are significant interspecific differences in the preferences of larval fish for phagostimulant types and concentrations. Furthermore, research on the mechanism of phagostimulant action in freshwater fish larvae remains fragmented, and a systematic application system has not yet been established.

The largemouth bass (*Micropterus salmoides*) is native to freshwater rivers and lakes in North America [19]. Due to its short growth cycle, strong disease resistance, wide optimal growth temperature range, and delicious meat, it has become one of the most important freshwater economic fish species in China [20]. However, the breeding of largemouth bass faces various challenges. Among the challenges in the largemouth bass larvae rearing, the current feed exhibits poor palatability, excessive reliance on costly live baits, low digestion and absorption efficiency, a predisposition to skeletal deformities, and high mortality rates, all of which lead to increased larvae rearing costs [21, 22]. Therefore, improving the feed utilization rate of the largemouth bass larvae and reducing the reliance on live baits have become urgent issues to be addressed.

At present, research on different attractants in adult and juvenile largemouth bass is available [23, 24], but there is a lack of studies on largemouth bass larvae. Therefore, this study aims to explore the effect of different attractants on the growth performance, air stress challenge, antioxidant capacity, immune system, and histology of the largemouth bass larvae. This study will provide valuable insights into the effective incorporation of different attractants into artificial feed and serve as a reference for further research on the nutrition of the largemouth bass larvae.

## 2. Materials and Methods

### 2.1. Diets and Experimental Design

TAU (99.00%), TMAO (97.00%), and DMPT (99.00%) were purchased from Shanghai Yuanye Biotechnology Co., Ltd., China. NTs (14.81%) were purchased from Shanghai Duwei Biotechnology Co., Ltd., China. Five isonitrogenous and isolipidic diets with different attractants were designed, and the four attractants were, respectively, added to the basal diet, and the dosages were determined based on previous research [3, 10, 25, 26]. All the five test diets were designed as follows: (1) control group (CON group, no attractants); (2) TAU group (supplement with 8 g/kg TAU); (3) TMAO group (supplement with 0.25 g/kg TMAO); (4)DMPT group (supplement with 5 g/kg DMPT); (5) NT group (supplement with 0.8 g/kg NTs). The specific formula is shown in Table 1. Fish meal, stickwater meal, and wheat protein hydrolysate were the main protein sources, while soy lecithin and fish oil served as the main fat sources. Fish meal, stickwater meal, wheat protein hydrolysate, and α‐starch were crushed and passed through a 60‐mesh sieve, while Ca(H_2_PO_4_)_2_ and trace elements were passed through an 80‐mesh sieve. The raw materials and different attractants were mixed evenly using the step‐by‐step expansion method, mixed in a V‐mixer for 20 min, then soy lecithin, fish oil, and water were added and mixed. The soft granule feed was made by an F‐26 twin‐screw extruder (South China University of Technology, Guangzhou, China) with a 1.5‐mm diameter die. After dried at 40°C in 24 h, it was crushed with a pulverizer and passed through 100, 80, 60, and 40‐mesh sieves. Then, it was stored at −20°C until use. The experimental feed formulation and nutrient composition are shown in Table 1.

**Table 1:** Composition and nutrient levels of experimental diets for largemouth bass larvae (as dry‐weight basis).

Ingredient (g/kg feed)	CON	TAU	TMAO	DMPT	NT
Fish meal	500	500	500	500	500
Stickwater meal	190	190	190	190	190
Wheat protein hydrolysate	80	80	80	80	80
α‐Starch	90	90	90	90	90
Soy lecithin	35	35	35	35	35
Fish oil	40	40	40	40	40
Vitamin mix^a^	5	5	5	5	5
Mineral mix^b^	5	5	5	5	5
Carboxymethyl cellulose	20	20	20	20	20
Choline chloride	5	5	5	5	5
Ca(H_2_PO_4_)_2_	20	20	20	20	20
Microcrystalline cellulose	10	2	9.75	5	9.2
Taurine (99.00%)	—	8	—	—	—
Trimethylamine oxide (97.00%)	—	—	0.25	—	—
Dimethyl‐β‐propiothetin (99.00%)	—	—	—	5	—
Nucleotides (14.81%)	—	—	—	—	0.8
Total	1000	1000	1000	1000	1000
Nutritional components					
Crude protein^c^ (%)	54.45	54.45	54.45	54.45	54.45
Crude lipid^c^ (%)	13.16	13.26	13.08	13.24	13.20
Ash^c^ (%)	13.98	14.08	14.22	14.19	14.36

^a^The vitamin premix provides per kilogram of feed: vitamin A, 16,000 IU; vitamin C, 150 mg; vitamin D3, 2000 IU; vitamin E, 180 mg; vitamin K3, 10 mg; vitamin B1, 16 mg; vitamin B2, 45 mg; vitamin B6, 20 mg; vitamin B12, 0.4 mg; calcium pantothenate, 70 mg; niacin, 80 mg; folic acid, 5 mg; biotin, 1 mg; inositol, 320 mg.

^b^The mineral premix provides per kilogram of feed: FeSO_4·_7H_2_O, 124.13 mg; CuSO_4_·5H_2_O, 9.77 mg; MnSO_4_·H_2_O, 26.15 mg; ZnSO_4_·7H_2_O, 154.53 mg; Na_2_SeO_3_, 0.44 mg; Ca(IO_3_)_2_, 2.31 mg; CoCl_2_·6H_2_O, 1.6 mg; MgSO_4_·7H_2_O, 1224.49 mg; zeolite powder 3456.59 mg.

^c^Crude protein, crude lipid, and ash given as the means of two determinations (*n* = 2).

Feed nutrient composition was analyzed by using standard methods of the Association of Official Analytical Chemists International [27]. Moisture was determined by drying in an oven at 105°C until constant weight. Crude protein was determined by the Kjeldahl method. Crude lipid was extracted by Soxhlet extraction with ether. Ash was determined by combustion in a muffle furnace at 550°C until constant weight.

### 2.2. Fish and Feeding Trial

The largemouth bass larvae were obtained from Nanxun Yuanliang Family Farm (Nanxun, China) and cultured in a recirculating water system in Huzhou University. Larvae derived from the largemouth bass broodstock of the same batch were used. Larvae were sent into each tank using a 250 mL stainless steel bowl and counted to ensure that the number of larvae in each tank was 5000. During this period, 100 fish were sampled, and the initial body weight (IBW) was measured and calculated using an analytical balance with a precision of 0.01% of the measured value (Hengping Balance Scientific Instrument Co., Ltd., Shanghai, China). The IBW was repeated three times to reduce errors. A total of 75,000 healthy larvae with an approximate size (1.25 ± 0.00 mg) were randomly divided into five groups with three replicates in black high‐density polyethylene tanks (40 cm × 60 cm × 80 cm). They were fed for 4 weeks. At the time of feeding, the light was turned on 30 min in advance, and the fish were fed to apparent satiety after gathering. Due to the small individual size of the experimental subjects and the difficulty in feed transition, a gradual transition feed acclimation method was adopted in this study based on production practices. This method involves sequentially adjusting the proportion of the target feed in the diet over a certain period, allowing the experimental subjects to gradually adapt to the new feed composition, thereby reducing stress responses caused by abrupt feed changes and improving the success rate of feed transition. In the first 7 days, the larvae were fed with the *Artemia* three times daily (at 07:00, 13:00, and 17:00). From 8th to 15th day, larvae were alternately fed with 80–100 mesh experimental feeds and *Artemia* six times daily (at 7:00, 7:30, 13:00, 13:30, 17:00, and 17:30), with feeds offered first followed by *Artemia*. A gradual replacement strategy was implemented, with *Artemia* being replaced by feeds at a daily rate of 12.5%, allowing larvae to adapt to the feeds progressively until the complete cessation of live bait feeding. As the fish grow and develop, different sizes of feed should be used. From 15th to 21th day, the larvae were fed 60–80 mesh experimental feeds; from 22nd to 25th day, they were fed 40–80 mesh experimental feeds, from 25th to 28th day, they were fed 20–40 mesh experimental feeds six times daily (7:00, 7:30, 13:00, 13:30, 17:00, and 17:30). During the feeding trial, the water quality conditions were as follows: dissolved oxygen was 5.5 ± 0.5 mg/L, water temperature was 26.5 ± 0.5°C, pH was 7.6 ± 0.6, ammonia nitrogen was no more than 0.5 mg/L, and nitrite nitrogen was no more than 0.1 mg/L.

### 2.3. The Hatching and Feeding of the *Artemia*

The *Artemia* used in the experiment were procured from Haixing County Hailin Aquatic Feed Co., Ltd. (Haixing, China). The hatching conditions for *Artemia* were as follows: salinity 30‰, pH 8–9, light intensity 2000 lx, dissolved oxygen 4–8 mg/L, and water temperature 29–30°C. Before each hatching, the frozen *Artemia* were taken out from −20°C in advance for preparation. An inverted pyramid‐shaped high‐density polyethylene bucket (15 L) was used for hatching *Artemia*. After 24 h of hatching, the *Artemia* were separated from the egg shells by darkening the container for 30 min, and then collected from the bottom of the bucket. After thorough cleaning to remove salt, the *Artemia* were poured into a plastic bucket (5 L) and filled with fresh water. A plastic dropper (50 mL) was used to stir and evenly distribute the feed, and full feeding was carried out.

### 2.4. Sample Collection and Preparation

After 21 days of the feeding trial, all the larvae in each tank were counted and weighed to determine weight gain (WG), specific growth rate (SGR), and SR, respectively. Growth performance of the largemouth bass larvae was calculated as follows:

Weight gain WG; %=100×Final weight−initial weight/initial weight.

Specific growth rate SGR; %/d=100×ln final weight−ln initial weight/days.

Survival rate SR; %=100×Final number of fish/initial number of fish.

### 2.5. Analysis of Activities of Antioxidant and Immunological Parameters

Four fish were randomly selected from each tank, quickly placed in liquid nitrogen, and subsequently stored at −80°C for antioxidant and immunological parameters. Total antioxidant capacity (T‐AOC), superoxide dismutase (SOD, A001‐1‐2), catalase (CAT, A007‐1‐1), malondialdehyde (MDA, A003‐1‐2), reduced glutathione (GSH, A006‐2‐1), acid phosphatase (ACP, A060‐2‐1), and alkaline phosphatase (AKP, A059‐2‐2) in whole fish were determined using commercial kits (Jiancheng Bioengineering Institute, Nanjing, China). The whole fish was weighed at 0.1 g, and 0.1 mol/L PBS at nine times the volume was added at a ratio of weight (g): volume (mL) = 1:9. Homogenization was performed at 60 Hz for 1 min using a tissue grinder (Jingxin Industrial Development Co., Ltd., Shanghai, China). The homogenate was centrifuged at 3500 rpm for 10 min, and the supernatant was collected for index determination.

### 2.6. Histological Analysis of the Intestine and Liver

Two fish were randomly selected from each tank. The whole fish was preserved in 4% formaldehyde solution and sent to make hematoxylin and eosin (H&E) stained sections for the intestine and liver (HaoKe Biotechnology Co., Ltd., Hangzhou, China). For each group, three intestine and liver slices were taken for analysis. The tissue was fixed in polymethylmethacrylate, embedded in paraffin using a paraffin embedding machine (Model JB‐L5, Wuhan Junjie Electronics Co., Ltd.), sectioned with a rotary microtome (Model HistoCore BIOCUT, Leica Microsystems Shanghai Co., Ltd.), and stained with H&E. Photographs of the intestine and liver were taken under a light microscope. The electronic images were analyzed using KFBIO Digital Slide Viewer software to assess various dimensions. In the intestinal slices, intestinal fold height, fold width, muscular layer thickness, and crypt depth were measured at magnification 50x, with three measurements per group. In the liver slices, the status of liver cells was observed at a magnification of 40x.

### 2.7. The Air Stress Challenge

To determine the feeding effect of the formulated feed and evaluate the quality of the larvae, an air stress challenge was conducted to assess their stress resistance. Thirty fish were randomly selected from each tank, and then after excess water was drained. They were placed on top of a flat, moist, fine‐mesh net (80 mesh). Continuous observation and recording were conducted. Fishes were considered dead if they stopped struggling and ceased breathing, and failed to recover after being placed back in water for a period of time (5 min). The time when all the largemouth bass larvae died was recorded.

### 2.8. Calculations and Statistical Methods

The normality of the data was assessed using the Shapiro–Wilk test. Prior to conducting one‐way analysis of variance (ANOVA), homogeneity of variances was evaluated for all datasets. The results were presented as mean ± standard deviation (SD) and subjected to ANOVA for statistical comparison. Post hoc comparisons among groups were performed using Duncan’s new multiple range test to determine significant differences. A probability level of *p* ≤ 0.05 was considered statistically significant. All statistical analyses were carried out using SPSS 27.0 (SPSS, Chicago, IL, USA).

## 3. Results

### 3.1. Growth Performance

The effects of different attractants in feed on the growth performance of largemouth bass larvae are shown in Table 2. Compared with the CON group, the final body weight (FBW), WG, and SGR were significantly increased in the TAU, DMPT, and NT groups (*p* < 0.05), with no significant differences observed among these three groups (*p* > 0.05). The SR was significantly increased in the TMAO, DMPT, and NT groups (*p* < 0.05), but no significant differences were observed among these three groups either (*p* > 0.05).

**Table 2:** Effects of different attractants on the growth performance of largemouth bass larvae (means ± SD, *n* = 3).

Index	CON	TAU	TMAO	DMPT	NT
IBW (mg)	1.2529 ± 0.0015	1.2529 ± 0.0015	1.2529 ± 0.0015	1.2529 ± 0.0015	1.2529 ± 0.0015
FBW (mg)	38.66 ± 2.50^b^	50.12 ± 3.88^a^	41.76 ± 3.38^b^	49.86 ± 3.04^a^	50.12 ± 3.59^a^
WG (%)	2990.43 ± 199.81^b^	3905.89 ± 310.44^a^	3238.06 ± 270.30^b^	3885.49 ± 243.31^a^	3905.92 ± 287.19^a^
SGR (%/day)	11.43 ± 0.22^b^	12.29 ± 0.26^a^	11.68 ± 0.28^b^	12.28 ± 0.20^a^	12.29 ± 0.24^a^
SR (%)	1.80 ± 0.18^c^	2.02 ± 0.38^c^	3.71 ± 0.31^a^	3.90 ± 0.33^a^	2.75 ± 0.27^b^

*Note:* Values with different superscript letters within the same row indicate significant differences (*p* < 0.05).

Abbreviations: FBW, final body weight; IBW, initial body weight; SGR, specific growth rate; SR, survival rate; WG, weight gain.

### 3.2. The Air Stress Challenge

**Figure 1:**
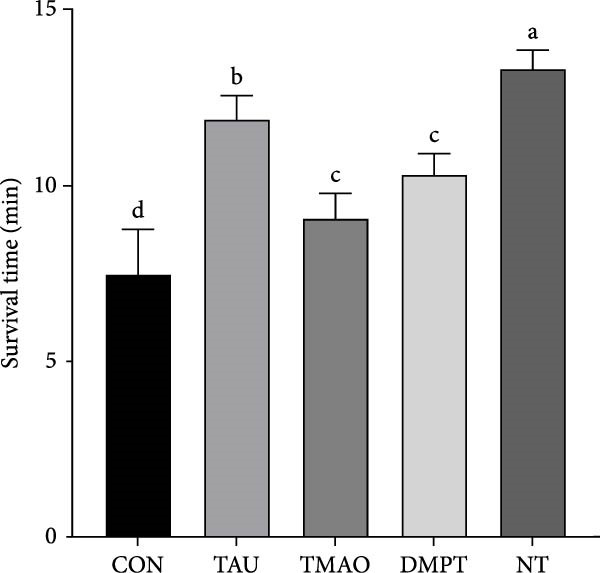
Effects of different attractants on the air stress challenge of largemouth bass larvae (means ± SD, *n* = 3). Values with distinct superscript letters within the same row indicate significant differences (*p* < 0.05).

The effects of different attractants in the diets on larvae under air stress challenge are shown in Figure 1. Compared with the CON group, the survival time was significantly increased in the TAU, TMAO, DMPT, and NT groups (*p* < 0.05). In addition, the survival time of the TAU and NT group was significantly longer than that of the other treatment groups (*p* < 0.05).

### 3.3. Antioxidant and Immunological Parameters

The effects of different attractants in the diets on antioxidant and immunological parameters are shown in Table 3. Compared with the CON group, the SOD was significantly increased in the TAU, DMPT, and NT groups (*p* < 0.05), and the NT group was significantly increased than that in the other treatment groups (*p* < 0.05). The content of MDA was significantly decreased in the TAU, TMAO, and NT groups (*p* < 0.05), but no significant differences were observed in MDA content among the three groups (*p* > 0.05). The CAT, ACP, AKP, and contents of GSH were significantly increased in the TAU, TMAO, DMPT, and NT groups (*p* < 0.05). Among them, the TAU and NT groups were significantly increased than the other treatment groups in the CAT and GSH; the NT group was significantly increased than the other treatment groups in the ACP; the DMPT group was significantly increased than the other treatment groups in the AKP. In addition, compared with the CON group and other treatment groups, the T‐AOC of the NT group was significantly increased (*p* < 0.05).

**Table 3:** Effects of different attractants on antioxidant and immunological parameters of largemouth bass larvae (means ± SD, *n* = 3).

Index	CON	TAU	TMAO	DMPT	NT
T‐AOC (U/mgprot)	0.41 ± 0.00^b^	0.42 ± 0.01^b^	0.43 ± 0.01^b^	0.42 ± 0.00^b^	0.46 ± 0.01^a^
SOD (U/mgprot)	32.77 ± 2.91^c^	40.04 ± 2.03^b^	35.82 ± 1.37^bc^	35.30 ± 0.73^bc^	46.58 ± 4.09^a^
MDA (nmol/gprot)	0.87 ± 0.02^a^	0.53 ± 0.06^b^	0.58 ± 0.06^b^	0.92 ± 0.02^a^	0.61 ± 0.06^b^
CAT (U/mgprot)	0.44 ± 0.03^c^	0.85 ± 0.05^a^	0.62 ± 0.11^b^	0.58 ± 0.06^b^	0.95 ± 0.07^a^
GSH (μmol/gprot)	2.26 ± 0.09^c^	2.63 ± 0.04^a^	2.45 ± 0.06^b^	2.50 ± 0.08^ab^	2.57 ± 0.06^ab^
ACP (U/gprot)	55.70 ± 3.58^d^	63.15 ± 2.79^c^	75.99 ± 3.16^b^	65.24 ± 1.58^c^	84.33 ± 3.09^a^
AKP (U/gprot)	64.05 ± 2.15^d^	72.40 ± 2.15^c^	85.40 ± 5.73^b^	114.75 ± 2.23^a^	86.46 ± 7.63^b^

*Note:* GSH, reduced glutathione. Values with distinct superscript letters within the same row indicate significant differences (*p* < 0.05).

Abbreviations: ACP, acid phosphatase; AKP, alkaline phosphatase; CAT, catalase; MDA, malondialdehyde; SOD, superoxide dismutase; T‐AOC, total antioxidant capacity.

### 3.4. Histology of the Intestine and Liver

The effects of different attractants in the diets on intestinal development are shown in Table 4 and Figure 2. Compared to the CON group, there was a significant increase in fold height, while muscular layer thickness, crypt depth, and fold width were significantly decreased in the TAU, TMAO, DMPT, and NT groups (*p* < 0.05). Specifically, the muscle layer thickness in the NT group was significantly lower than that in the other treatment groups (*p* < 0.05); the crypt depth in the TMAO group was significantly lower than that in the other treatment groups, while its fold height was significantly greater than that in the other treatment groups (*p* < 0.05); the fold width in the DMPT group was significantly lower than that in the other treatment groups (*p* < 0.05).

Figure 2Intestinal H&E‐stained sections (magnification 50x; I: muscular layer thickness, Ⅱ: crypt depth, Ⅲ: fold height, and Ⅳ: fold width). (A–E) The subparts indicate largemouth bass larvae fed a basal diet, different attractants with 0.8% taurine (99.00%), 0.025% trimethylamine oxide (97.00%), 0.5% dimethyl‐β‐propiothetin (99.00%), and 0.08% nucleotides (14.81%), respectively.

**Figure:**
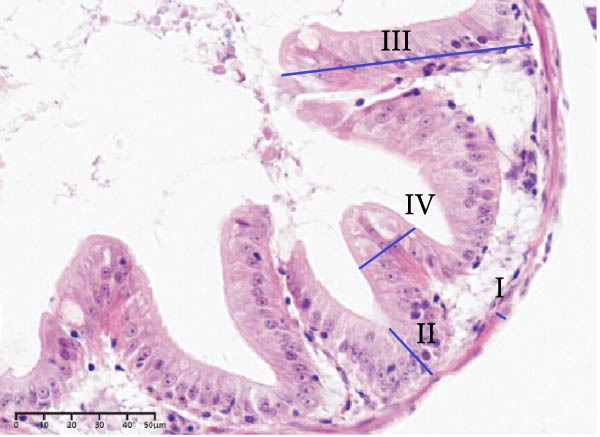
(A)

**Figure:**
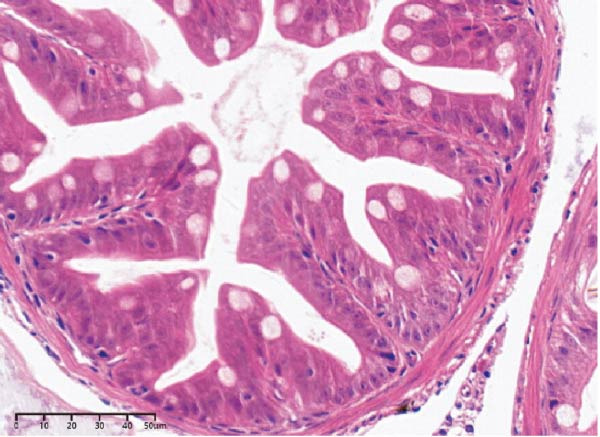
(B)

**Figure:**
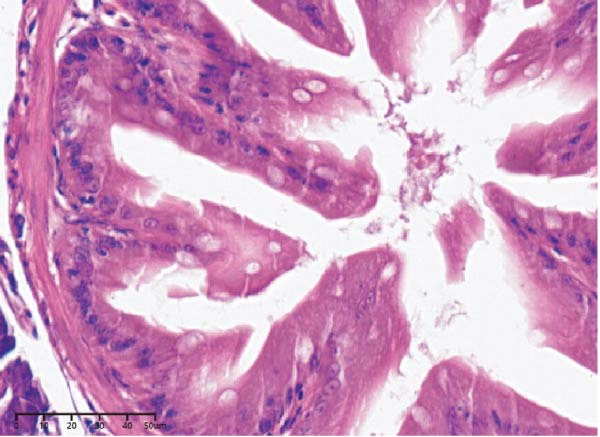
(C)

**Figure:**
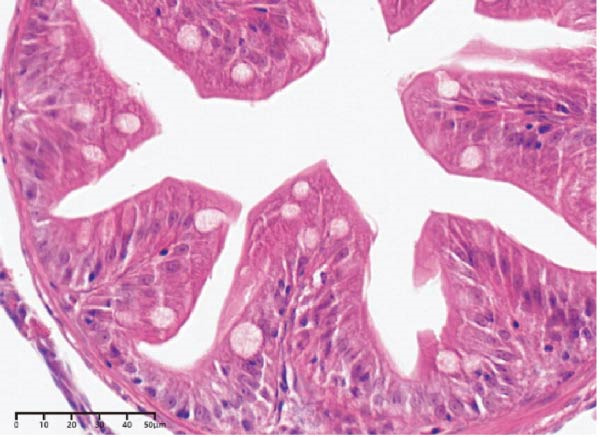
(D)

**Figure:**
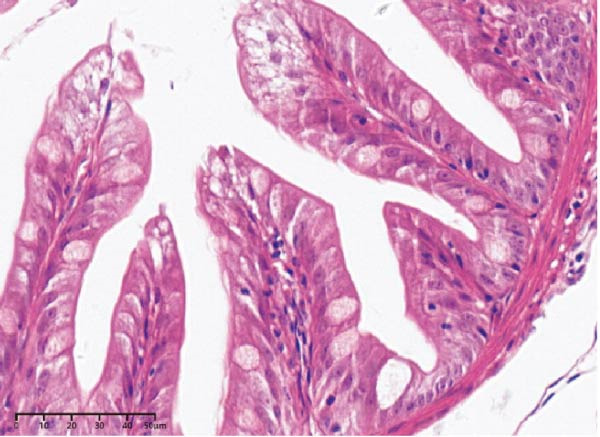
(E)

**Table 4:** Effect of different attractants on intestinal development of largemouth bass larvae (means ± SD, *n* = 3).

Index	CON	TAU	TMAO	DMPT	NT
Muscular layer thickness (μm)	11.26 ± 0.76^a^	8.15 ± 0.29^b^	7.52 ± 0.45^bc^	8.02 ± 0.54^b^	7.02 ± 0.58^c^
Crypt depth (μm)	24.79 ± 1.42^a^	17.02 ± 0.88^b^	14.57 ± 1.12^c^	17.04 ± 1.55^b^	16.26 ± 1.47^bc^
Fold height (μm)	80.65 ± 7.56^c^	82.89 ± 2.59^c^	116.87 ± 10.58^a^	98.89 ± 7.86^b^	100.82 ± 5.78^b^
Fold width (μm)	35.34 ± 0.88^a^	31.39 ± 1.77^b^	30.01 ± 1.40^b^	28.13 ± 0.86^c^	30.13 ± 0.89^b^

*Note:* Values with distinct superscript letters within the same row indicate significant differences (*p* < 0.05).

The effects of different attractants in the diets on liver sections stained by H&E are shown in Figure 3. Compared to the CON group and other treatment groups, the TMAO and NT groups alleviated the hepatocyte enlargement, loss of nuclei, and cytoplasmic vacuolization.

Figure 3Liver H&E‐stained sections (magnification 40x). (A–E) The subparts indicate largemouth bass larvae fed a basal diet, different attractants with 0.8% taurine (99.00%), 0.025% trimethylamine oxide (97.00%), 0.5% dimethyl‐β‐propiothetin (99.00%), and 0.08% nucleotides (14.81%), respectively. “H” indicates hepatocyte enlargement, “L” indicates loss of nuclei, and “C” indicates cytoplasmic vacuolization.

**Figure:**
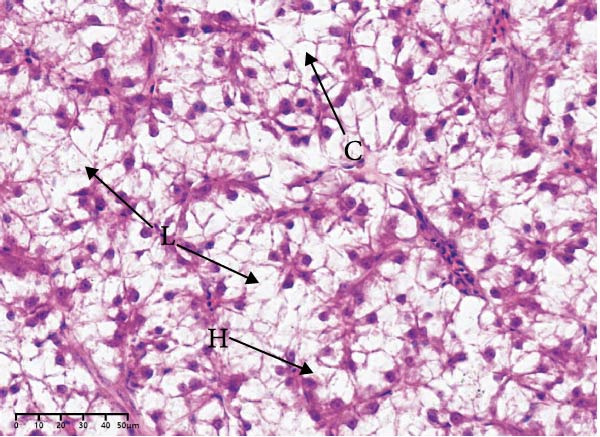
(A)

**Figure:**
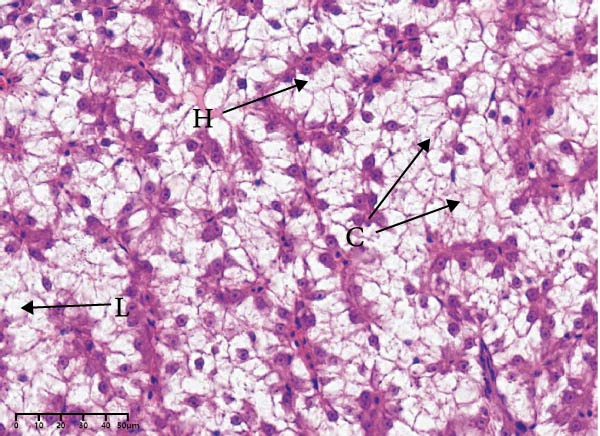
(B)

**Figure:**
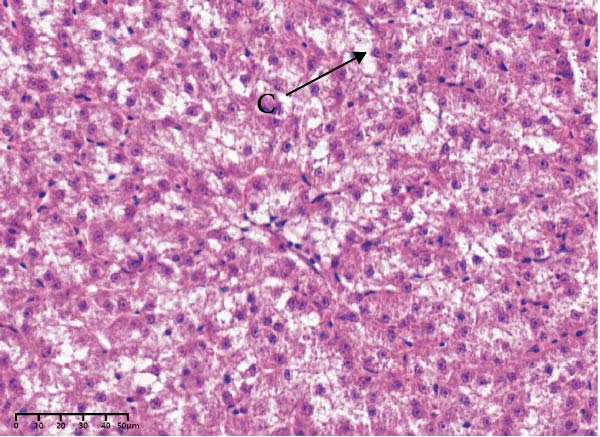
(C)

**Figure:**
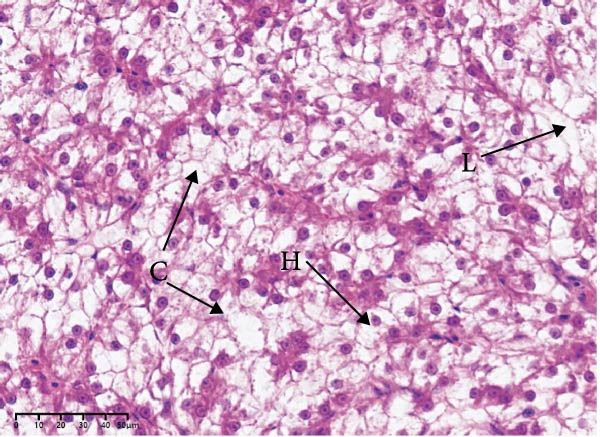
(D)

**Figure:**
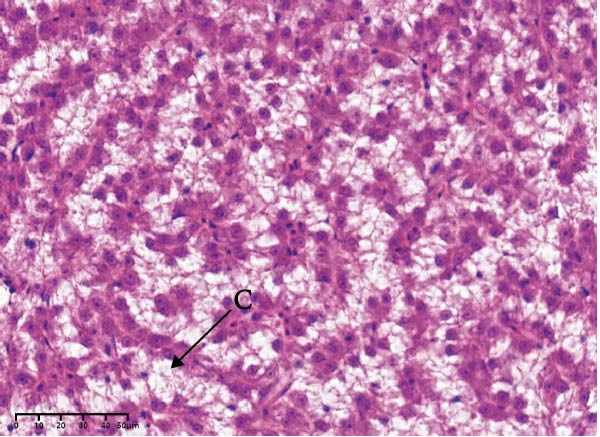
(E)

## 4. Discussion

Currently, research related to feeding attractants has mostly focused on the domestication process of special aquatic fish species, as well as the stages of adult fish and large‐sized larvae. However, studies on small‐sized larvae, especially during the critical period of food transition, have been relatively scarce due to issues such as high mortality rates encountered in experimental processes. Against this backdrop, this study aims to investigate the effects of different feeding attractants on the growth performance, antioxidant capacity, immune, and intestinal and hepatic histology of largemouth bass larvae.

In this study, we found that supplementing TAU, DMPT, and NT in the initial feed of largemouth bass larvae significantly increased the FBW, WG, and SGR, while supplementing TMAO, DMPT, and NT significantly improved the SR. These findings are similar to previous research results. TAU is highly concentrated in animal tissues, especially in the heart, retina, skeletal muscle, brain, large intestine, plasma, blood cells, and white blood cells [28]. Therefore, this conditionally essential amino acid plays an important role in many physiological functions, including membrane stabilization, antioxidation, detoxification, regulation of immune response, calcium transport, myocardial contractility, retinal development, bile acid metabolism, osmoregulation, and endocrine function [29]. Hongmanee et al. [30] demonstrated in a 60‐day feeding trial on juvenile snakehead fish (*Channa striata*) that supplementing TAU in the fish diet could effectively increase the WG, average daily gain (ADG), and SGR, but had no significant effect on SR. Similar results were observed in this study, that TAU increased FBW, WG, and SGR, but did not improve SR. The functions of TAU are concentrated in areas such as growth metabolism, antioxidant defense, and osmoregulation, and its impact on survival is indirectly auxiliary rather than directly protective [31]. Supplementing TMAO in the diet increased SR but did not significantly increase WG, which may be partly attributed to the different adaptability of different fish species to TMAO. For instance, adding 0.025% TMAO to the diet of taimen (*Hucho taimen*) affected the WG, but adding TMAO to the diet of rainbow trout (*Oncorhynchus mykiss*) had no significant effect on the WG, feed conversion ratio (FCR), and feed intake (FI) [5, 10]. Supplementing DMPT and NT in the feed, DMPT mainly improves the feeding efficiency of fish by stimulating their olfaction, while NT can enhance the nitrogen metabolism level of fish and improve the protein utilization efficiency. Furthermore, exogenous supplementation of NT can promote cell division and differentiation. Therefore, they can both promote the growth performance of fish and increase the SR [3, 4]. Liu et al. [32] supplemented 260 mg/kg DMPT in the all‐plant protein diet of grass carp (*Ctenopharyngodon idella*) and improved the growth performance of grass carp and reduced the damage to the intestine caused by the all‐plant protein diet. Guo et al. [33] supplemented 90 mg/kg NT in the basal diet of white shrimp (*Litopenaeus vannamei*) and effectively increased the growth performance, such as WG and SR, but indicated that the promoting effect of NT on growth performance might be short‐term. Therefore, adding attractants to the initial feed, each attractant can promote the growth performance of largemouth bass larvae from different physiological and biochemical perspectives, and have a significant increase in the WG, SGR, and SR.

It is well known that the antioxidant capacity indicators of fish are closely related to the growth performance. Antioxidant enzyme systems such as SOD and CAT can effectively remove free radicals in the body, while nonenzymatic antioxidants such as GSH participate in regulating the redox balance [34, 35]. In addition, the content of MDA reflects the degree of oxidative damage to cells, and the T‐AOC comprehensively reflects the antioxidant defense level of the body [36]. When fish are under stress, abnormal antioxidant indicators may cause oxidative stress, leading to cell damage and metabolic disorders, which can inhibit their growth. On the contrary, good antioxidant capacity helps maintain the physiological homeostasis of the organism, reduces oxidative damage, and improves the utilization efficiency of nutrients, thereby promoting growth performance [37]. Therefore, antioxidant parameters are important for evaluating the growth potential and health status of fish. The results of this study showed that supplementing TAU and NT in the diet of largemouth bass larvae can significantly increase the activities of SOD, CAT, and GSH, and significantly decrease the content of MDA. At the same time, this finding is similar to the results of the air stress challenge. The largemouth bass larvae supplemented with TAU and NT had significantly longer survival time than the CON and the other treatment groups when exposed to air. Teles et al. [38] found that supplemented the diet of juvenile almaco jack (*Seriola rivoliana*) with 2% TAU, thereby enhancing the antioxidant parameters. Additionally, Victor et al. [39] added 5 g/kg of NT to the basal diet of largemouth bass larvae, effectively enhancing the activities of antioxidant enzymes in the serum and liver. In the diet of larval fish, SOD activity was significantly increased after supplementing TMAO, but the MDA content did not show a significant change; while in the case of supplementing DMPT, the SOD activity was not significantly different from the CON, but the MDA content was significantly decreased. This may be due to the time lag effect in protein modification, gene transcription, and translation processes, resulting in inconsistent results [40]. Moreover, supplementing NT in the diet of largemouth bass larvae can also increase the T‐AOC. Similar results have also been described in various fish species fed with NTs‐supplemented diets, such as grass carp [41], yellow catfish (*Pelteobagrus fulvidraco*) [42], rainbow trout [43], large yellow croaker [18], and sterlet sturgeon (*Acipenser ruthenus*) [44]. In summary, supplementing different attractants in the diet of largemouth bass larvae has different promoting effects on the antioxidant capacity, with NT and TAU showing better results.

The enhancement of fish immunity through feeding attractants is the result of a “superposition of multiple benefits.” On one hand, feeding attractants promote food intake to ensure nutrient supply, thereby providing a material basis for immune function. For example, they enhance food intake by boosting the activity of digestive enzymes and the expression of appetite‐related genes, which in turn improves immune capacity [18]. On the other hand, the active components of some feeding attractants directly regulate immune function. For instance, TAU reacts with hypochlorous acid produced by neutrophils to form TAU chloramine, which modulates inflammatory responses (inhibiting the production of pro‐inflammatory factors and regulating immune cell activity) [45]. It also reduces oxidative damage to immune cells caused by reactive oxygen species by stabilizing mitochondrial function, while maintaining red blood cell membrane stability and osmotic pressure to alleviate the immune burden induced by hemolysis, thereby directly promoting fish immunity [46]. Meanwhile, the improvement of feeding behavior and the optimization of the aquaculture environment reduce stress and pathogen pressure, further alleviating the immune burden on fish. The ACP and AKP serve as nonspecific immune parameters in fish, with their activities being closely associated with growth performance [47]. ACP mainly participates in the digestion and decomposition of pathogens by phagocytes through lysosomes, and regulates inflammatory responses and cell apoptosis; while AKP is widely distributed on the cell membrane surface, participating in the regulation of substance metabolism and playing an important barrier role in intestinal mucosal immunity [48, 49]. When the activities of ACP and AKP are at normal levels, the immune capacity of the organism is enhanced, thereby reducing the risk of pathogen infection and avoiding excessive energy consumption due to excessive immune responses, which allows nutrients to be more efficiently utilized for growth performance [36]. In addition, AKP can also assist in the hydrolysis of phosphoester nutrients in the intestine, improving digestion and absorption efficiency [50]. This study shows that four attractants can significantly increase the immune parameters of largemouth bass larvae. Previous studies have also reached similar conclusions. For instance, Dong et al. demonstrated that supplementing 0.4% and 0.8% TAU could effectively enhance the activities of AKP and ACP in the serum of Chinese mitten crab (*Eriocheir sinensis*) [51]; Fang et al. found that adding 0.06% DMPT to the diet of Yellow River carp (*Cyprinus carpio* var) could effectively increase the activity of ACP [52]; in addition, Guo et al. reported that adding 90 mg/kg NT to the feed of whiteshrimp significantly increased the activity of ACP in the serum [33]; however, present research on the impact of TMAO on the immune parameter of fish or shrimp is still limited. In summary, it can be seen that different attractants can improve the immune parameters of largemouth bass larvae. Additionally, NT is superior to other attractants in improving the ACP, and the addition of DMPT is superior to other attractants in enhancing the AKP.

In terms of intestinal histology, such as fold width, fold height, muscular layer thickness, and crypt depth, they are considered important for the nutrient absorption of fish. The intestinal histology in this study showed that adding four attractants to the diet of largemouth bass larvae could decrease the muscular layer thickness, crypt depth, and fold width, while increasing fold height. Zhu et al. [53] found that adding 10 g/kg TAU to the diet of juvenile *Rhynchocypris lagowskii* Dybowski with an 80 g/kg glycinin diet effectively alleviated the damage to the intestine caused by an all‐plant diet. Ma et al. [54] reached a similar conclusion in the feeding trial of yellow drum (*Nibea albiflora*). Bockus et al. [5] found that adding 10 g/kg TMAO to the diet of rainbow trout effectively alleviated the intestinal damage caused by soybean meal. Adding 260 mg/kg DMPT to an all‐plant diet of grass carp effectively alleviated the damage to the intestinal tissue caused by the diet [32]. Adding 30 g/kg yeast supplement rich in NT to the daily diet of white shrimp effectively increased fold height and microvillus height [55]. In summary, supplementing four attractants in the diet of largemouth bass larvae can effectively improve intestinal tissue and promote fold development in the intestine, with TMAO showing better results. However, current research on the underlying physiological and biochemical mechanisms by which TMAO regulates intestinal tissue improvement remains relatively scarce, and its specific action pathways await further in‐depth investigation.

In this study, the results of the liver histological study indicated that TAU and DMPT had no significant promoting effect on the liver of larvae. In addition, this is consistent with the results of a recent study on juvenile snakehead fish (*C. striata*), which pointed out that there were no significant differences in liver histological structure when the diet was supplemented with 0.5%, 1.0% and 1.5% TAU [30]. Moreover, TMAO and NT had a promoting effect on the liver of larvae. A recent study also obtained similar results, finding that adding 500 mg/kg NTs to the diet of European seabass could alleviate mild hepatocytic cloudy swelling and congestion of main blood vessels [56]. Currently, research on the effects of dietary TMAO and DMPT supplementation on fish liver remains relatively scarce, and the underlying physiological and biochemical mechanisms by which feeding attractants regulate liver histology await further in‐depth investigation.

## 5. Conclusion

In conclusion, the results showed that four attractants have positive effects on the growth performance, antioxidant capacity, immunity, and histology study of largemouth bass larvae. The promotion of growth performance by supplementing TAU, TMAO, DMPT, and NT in the feed due to the special odor or the improvement of the metabolic level in fish. Even more significantly, the improvement of growth performance cannot be separated from excellent antioxidant capacity, immunity, air stress challenge, intestinal development, and liver cell status. Adding attractants to the feed of largemouth bass larvae can optimize their supplementation effects in fish diets, and thereby increase aquaculture production. However, the underlying mechanisms and optimal addition level still require further research.

## Disclosure

All authors read and approved the final manuscript.

## Conflicts of Interest

The authors declare no conflicts of interest.

## Author Contributions

All authors contributed to the study conception and design. Conceptualization, methodology, and writing – original draft were formulated and written by Jianle Yang. Funding acquisition, supervision, project administration, and resources were led by Qiyou Xu. Participated in the sample collection was worked by Jianle Yang, Haoze Wang, Xiaorui Fan, Jiaqi Wang. Writing – review and editing were finished by Jianle Yang, Qiyou Xu, and Jianhua Zhao, and all authors commented on previous versions of the manuscript.

## Funding

This work was supported by the “Pioneer” and “Leading Goose” R&D Program of Zhejiang under grant number [2023C02024].

## Data Availability

The article contains the original contributions made in this study, and readers may direct further inquiries to the corresponding author.
